# Supplementary material for: Patients’ Post-/Long-COVID Symptoms, Vaccination and Functional Status—Findings from a State-Wide Online Screening Study
Source: Vaccines (Basel). 2023 Mar 17;11(3):691. doi: 10.3390/vaccines11030691 (PMC10051253; doi:10.3390/vaccines11030691)
Supplement: Supplementary file 1 [file vaccines-11-00691-s001.zip › vaccines-2191664-supplementary.pdf]

Table S1 *Descriptives for vaccination status in detail*

	Gender:<br>Female<br>Male	Vaccination …<br>Before<br>During<br>After… the infection	Age *M (SD)*	BMI
No vaccination	5 (62.5%)<br>3 (37.5%)	-	55.57 (4.47)	29.66 (4.87)
One vaccination	4 (80.0%)<br>1 (20.0%)	2 (40.0%)<br>3 (60.0%)<br>0 (0.0%)	38.20 (17.68)	25.20 (2.34)
Two vaccination	30 (68.2%)<br>14 (31.8%)	19 (42.2%)<br>24 (53.3%)<br>2 (4.4%)	43.09 (12.27)	27.18 (5.88)
≥ 3 vaccinations	88 (73.3%)<br>32 (26.7%)	20 (16.8%)<br>90 (75.6%)<br>9 (7.6%)	44.58 (11.75)	27.11 (5.88)
No indication	34 (77.3%)<br>10 (22.7%)	1 (50%)<br>1 (50%)<br>0 (0.0%)	44.34 (10.66)	30.00 (12.93)
Total	*161 (72.9%)*<br>*60 (27.1%)*	*43 (25.0%)*<br>*118 (68.6%)*<br>*11 (6.4%)*	*44.43 (11.77)*	*27.30 (6.27)*
Statistics	$Chi^2(4)=1.497$; $p=.827$	Due to too small cell sizes: could not be computed	$F(4,216)=2.11$; $p=.080$	$F(4,155)=0.683$; $p=.605$

Note. Varying sample sizes and missings are due to not reported gender, vaccination status, age or BMI.

Table S2

Descriptives of the repeated measurement ANOVA to examine RQ2

	Before vs. after/during the vaccination	*M*	*SD*	*N*
Symptoms at T1	Before	2.8429	0.60401	34
	During/after	2.6310	0.56095	103
	Total	2.6836	0.57704	137
Symptoms at T2	Before	2.3739	0.50693	34
	During/after	2.3760	0.59945	103
	Total	2.3755	0.57607	137
Symptoms at T3	Before	2.3372	0.45909	34
	During/after	2.3218	0.60145	103
	Total	2.3256	0.56789	137

Table S3

Descriptives of the MANOVA to examine RQ3

			M	*SD*	*N*
Participation	No vaccination	female	4.3902	0.03750	2
		male	2.8750	1.47314	2
		total	3.6326	1.22028	4
	One vaccination	female	3.4375	0.92390	4
		male	3.6667	-	1
		total	3.4833	0.80666	5
	Two vaccination	female	3.6107	0.54985	26
		male	3.4097	0.85610	12
		total	3.5472	0.65661	38
	≥ 3 vaccinations	female	3.2852	0.71720	75
		male	3.5914	0.99124	30
		total	3.3727	0.81197	105
	No indication	female	2.3485	0.29742	4
		male	3.0625	1.45516	4
		total	2.7055	1.04454	8
	Total	female	3.3531	0.71819	111
		male	3.4760	0.98925	49
		total	3.3907	0.80963	160
Symptoms	No vaccination	female	2.3929	0.45457	2
		male	2.2500	0.35355	2
		total	2.3214	0.34256	4
	One vaccination	female	2.6429	0.96362	4
		male	1.7143	-	1
		total	2.4571	0.93214	5
	Two vaccination	female	2.3324	0.49112	26
		male	2.3819	0.65948	12
		total	2.3480	0.54112	38
	≥ 3 vaccinations	female	2.3820	0.54080	75
		male	2.3452	0.59617	30
		total	2.3715	0.55451	105
	No indication	female	2.5893	0.68852	4
		male	1.9643	0.48269	4
		total	2.2768	0.64392	8
	Total	female	2.3874	0.54432	111
		male	2.3063	0.59255	49
		total	2.3626	0.55891	160

Workability	No vaccination	female	8.50	0.707	2
		male	9.00	1.414	2
		total	8.75	0.957	4
	One vaccination	female	5.50	3.109	4
		male	3.00	-	1
		total	5.00	2.915	5
	Two vaccination	female	6.00	2.040	26
		male	4.83	2.082	12
		total	5.63	2.098	38
	≥ 3 vaccinations	female	4.79	2.321	75
		male	4.57	2.029	30
		total	4.72	2.234	105
	No indication	female	2.50	1.732	4
		male	7.25	2.363	4
		total	4.87	3.182	8
	Total	female	5.08	2.375	111
		male	5.00	2.273	49
		total	5.06	2.337	160
Life satisfaction	No vaccination	female	2.7500	0.70711	2
		male	2.7500	1.41421	2
		total	2.7500	0.91287	4
	One vaccination	female	2.5000	0.40825	4
		male	2.7500	-	1
		total	2.5500	0.37081	5
	Two vaccination	female	2.4872	0.44826	26
		male	2.2917	0.59193	12
		total	2.4254	0.49842	38
	≥ 3 vaccinations	female	2.0544	0.58598	75
		male	2.1250	0.75358	30
		total	2.0746	0.63538	105
	No indication	female	1.6875	0.47324	4
		male	1.8125	0.55434	4
		total	1.7500	0.48181	8
	Total	female	2.1712	0.58591	111
		male	2.1786	0.72169	49
		total	2.1734	0.62828	160

Note. – indicates that no SD could be computed.
